# Levodopa affects spike and local field synchronisation in the pedunculopontine nucleus of a rat model of Parkinson’s disease

**DOI:** 10.18632/aging.202585

**Published:** 2021-02-26

**Authors:** Hao Zhang, Jinlu Xie, Yaqiong Li, Huimin Liu, Chuanguo Liu, Dongfang Kan, Xiwen Geng, Sheng Wei

**Affiliations:** 1 Experimental Centre, Shandong University of Traditional Chinese Medicine, Ji’nan 250355, Shandong, China; 2 Key Laboratory of Vector Biology and Pathogen Control of Zhejiang, School of Medicine, Huzhou University, Huzhou Central Hospital, Huzhou 313000, Zhejiang, China; 3 Department of Traditional Chinese Medicine, Shandong University of Traditional Chinese Medicine, Ji’nan 250355, Shandong, China

**Keywords:** local field potential, Parkinson’s disease, coherence, pedunculopontine nucleus, spike

## Abstract

The pedunculopontine nucleus (PPN) undergoes significant anatomic and electrophysiological alterations in Parkinson’s disease (PD), severely impacting locomotion. However, the effect of 6-hydroxydopamine (6-OHDA) lesion and levodopa (L-DOPA) therapy on the relationships between spike activities and local field potential (LFP) within the PPN is not well-understood. Synchronisation between the spike activity of individual neurones and LFP of neuronal ensembles is a crucial problem in the pathogenesis of PD. In this study, LFP signals and spikes in the PPN of rats in control, lesioned, and L-DOPA groups were recorded synchronously with a multi-unit electrical signal acquisition system and analysed for their coherence value, spike-field coherence, and phase-lock relationship. The spike-LFP relationship in the PPN was markedly increased in specific frequency bands because of the 6-OHDA lesion but differed depending on the animal locomotion state and neuronal type. L-DOPA had a limited therapeutic effect on the 6-OHDA-induced increase in the coherence value. Our study demonstrates that the PPN spike-LFP relationship is involved in the pathogenesis of PD and is critical for the effects of L-DOPA, providing a basis for the clinical treatment of refractory PD symptoms.

## INTRODUCTION

Parkinson’s disease (PD) is a common neurodegenerative disorder characterised by progressive motor and nonmotor disability [1]. Pathologically, PD is caused by the loss of dopamine production by dopaminergic neurones of the substantia nigra pars compacta (SNc), which is the primary source of dopaminergic projections to the striatum [2].

Currently, no disease-modifying treatments are available for PD. Since its introduction in the 1960s, levodopa (L-DOPA) has been the first-line and most effective clinical treatment for PD because it can improve most motor features by restoring dopaminergic activity in the striatum [3, 4]. However, some axial symptoms, such as severe gait and postural impairments, are not ameliorated by L-DOPA; however, the reasons for this are unclear. The pedunculopontine nucleus (PPN) region has attracted attention, as deep brain stimulation (DBS) in the PPN was reported as a promising therapy for axial motor deficits in PD, particularly gait and postural impairments [5, 6].

The heterogeneous neuronal population of the PPN reportedly forms the neuroanatomical basis of the midbrain locomotor region, with an important role in the initiation, acceleration, deceleration, and termination of locomotion [7, 8]. Important connections exist between the PPN and cortico-basal ganglia loop [8, 9]. Furthermore, the PPN exhibits significant histological and physiological alterations in PD. In patients with PD, significant neuronal loss is observed in the PPN [10] and neuronal spiking activities are altered in two distinct disease processes [11]. Moreover, several studies revealed changes in the electrophysiological characteristics of the PPN in PD animal models and in patients [12–15]. Moreover, our previous studies demonstrated an increased firing rate and altered local field potential (LFP) oscillations in the PPN of a rat PD model based on 6-hydroxydopamine (6-OHDA)-induced lesions [16, 17]. The above evidence supports the existence of a close causal relationship between the neuronal activity of PPN and PD.

Synchronised recording and analysis of spike activity in individual neurones and the LFP of neurone ensembles (spike-LFP synchronisation) are widely to evaluate neuronal function [18–20]. However, the impact of PD pathogenesis and L-DOPA therapy on the spike-LFP synchronisation of PPN neurones is unclear. To determine the role of PPN in gait and posture functions and clinical symptoms refractory to L-DOPA therapy, studies are needed to examine PPN neuronal activities after 6-OHDA and L-DOPA treatment.

**Figure 1:**
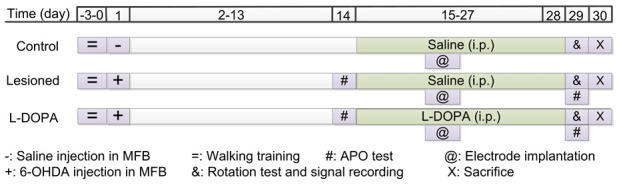
**Representation of the experimental timeline.** MFB: medial forebrain bundle; APO: apomorphine; 6-OHDA: 6-hydroxydopamine.

In this study, we simultaneously recorded the spontaneous discharge of individual neurones and LFP activities in the PPN after implantation of multichannel electrodes in control, 6-OHDA-lesioned, and L-DOPA-treated rats under resting conditions or specific locomotor states and analysed the spike-LFP coherence value, spike-field coherence (SFC), and phase-lock relationship. The experimental timeline is shown in Figure 1.

## RESULTS

### Loss of dopaminergic neurones in the SNc following the 6-OHDA lesion

**Figure 2:**
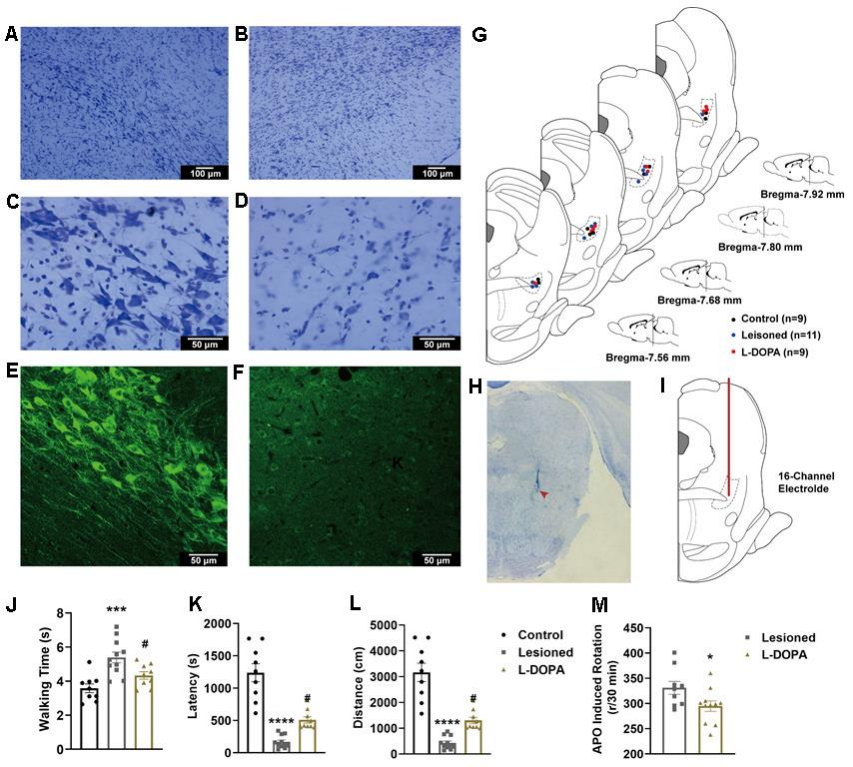
(**A**–**D**) Exemplar images of cresyl violet-stained coronal sections of the substantia nigra pars compacta of animals with 6-hydroxydopamine (6-OHDA) lesions. (**A**) control hemisphere, 100×; (**B**) lesioned hemisphere, 100×; (**C**) control hemisphere, 400×; (**D**) lesioned hemisphere, 400×. (**E**, **F**) fluorescence immunohistochemical staining of dopaminergic neurones for tyrosine hydroxylase in the substantia nigra pars compacta. (**E**) Control hemisphere, 200×; (**F**) lesioned hemisphere, 200×. (**G**) Schematic representation of electrode positioning in rats of different groups. (**H**) Image of a coronal section of PPN stained with cresyl violet. The location of the electrode tip is indicated by a red arrowhead. (**I**) Schematic representation of the electrode path (red line) into the pedunculopontine nucleus (PPN). (**J**) Walking time along a control ladder in lesioned and levodopa (L-DOPA) rats. (**K**) Latency in the rotarod test. (**L**) distance in the rotarod test. (**M**) Number of apomorphine (APO)-induced rotations after 30 min in lesioned and L-DOPA rats. * p < 0.05, *** p < 0.001, **** p < 0.0001 compared to the control group; # p < 0.05 compared to the lesioned group.

Neurone loss in SNc following the 6-OHDA lesion was confirmed by cresyl violet and TH immunohistochemical staining. As shown in Figure 2A–2F, TH-positive neurones were significantly depleted in the SNc ipsilateral to the 6-OHDA lesion. Cresyl violet staining revealed that remnant neurones on the lesioned side displayed altered morphology compared to those on the control side. Histological analysis showed severe 6-OHDA-induced lesion of SNc dopaminergic neurones, confirming successful establishment of the disease model.

### L-DOPA treatment improved locomotion in animals with PD

In this study, only rats with correct electrode positioning were used for analyses. A total of 9 control, 11 lesioned, and 9 L-DOPA rats were examined. For these animals, cresyl violet-stained PPN sections with electrode tip sites are shown in Figure 2G. An image of a coronal slice as well as the electrode path are shown in Figure 2H, 2I.

The walking time along the ladder, latency, distance in the rotarod test, and APO-induced rotations after 30 min were calculated to assess locomotor capacity. In the walking test shown in Figure 2J (F (2,26) = 11.21, p = 0.0003 (one-way ANOVA test)), lesioned rats required more time to walk along the ladder compared to control rats (p = 0.0002). This effect was reversed upon L-DOPA treatment (p = 0.0290). In the rotarod test shown in Figure 2K, 2L (F (2,26) = 40.94, p < 0.0001 for latency; F (2,26) = 40.93, p < 0.0001 for distance), the shorter latency (p < 0.0001) and distance (p < 0.0001) of the lesioned animals were improved upon L-DOPA treatment (p = 0.0256 and 0.0256, respectively). Animals in the L-DOPA group showed fewer APO-induced rotations (Figure 2M) than rats in the lesioned group (t = 2.225, df = 18, p = 0.0391). These results indicate that L-DOPA treatment significantly improved the locomotor behaviour of animals with experimental PD.

### Neuronal classification in PPN

**Figure 3:**
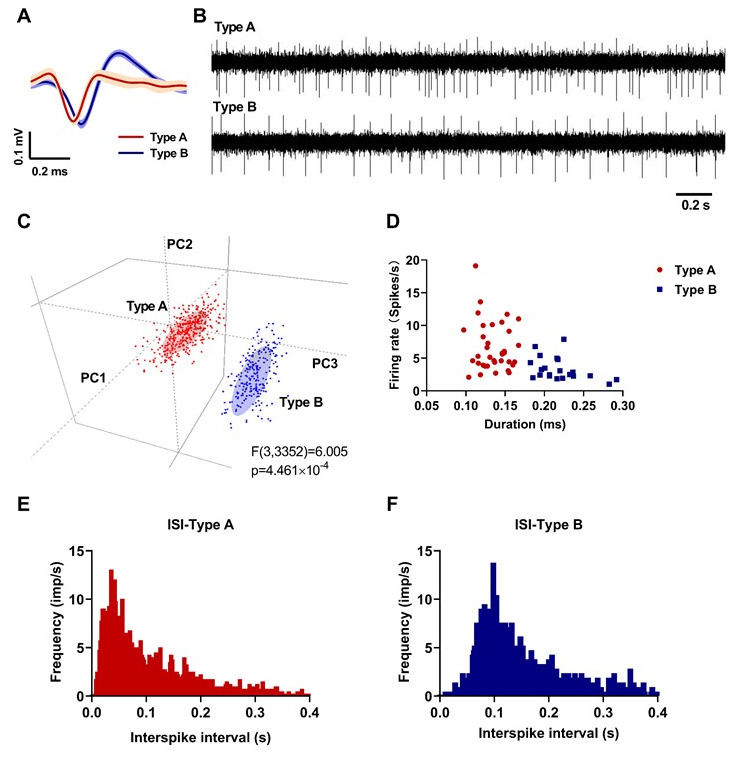
**Neuronal classification results in the pedunculopontine nucleus (PPN) (red: Type A; blue: Type B).** (**A**) Averaged waveforms of each neuronal type. (**B**) Representative signals of each neuronal type. (**C**) Principal component analysis (PCA) results in 3D view. (**D**) Spike duration (trough-to-peak) and firing rate of 36 Type A and 21 Type B neurones. (**E**) Representative inter-spike interval histogram of a Type A neurone. (**F**) Representative inter-spike interval histogram of a Type B neurone.

From the recordings of nine control rats in a resting state, 57 neurones were collected and classified into two distinct categories (36 Type A and 21 Type B) according to the duration (trough-to-peak) and shape of the waveforms and firing properties. As shown in Figure 3, Type A neurones exhibited a narrower duration with a “V” shape waveform (Figure 3A), higher firing rate, and more irregular firing pattern than Type B neurones (Figure 3B, 3D). From the principal component analysis (PCA) results in 3D view (two neurone types recorded by one electrode channel) in Figure 3C, the two neurone types showed a distinct distribution according to the three main components (F (3,3352) = 6.005, p < 0.0001). Moreover, as shown by the inter-spike intervals (ISI) histograms (Figure 3E, 3F), Type A neurones showed a Poissonian tonic distribution and Type B neurones showed a concentrated distribution, respectively, indicating distinct firing patterns. The results of PPN neurone classification in lesioned and L-DOPA-treated rats in the resting and locomotion state were as described above (data not shown).

### Effect of L-DOPA on the association between spiking activities and LFP in the PPN

### Coherence value

**Figure 4:**
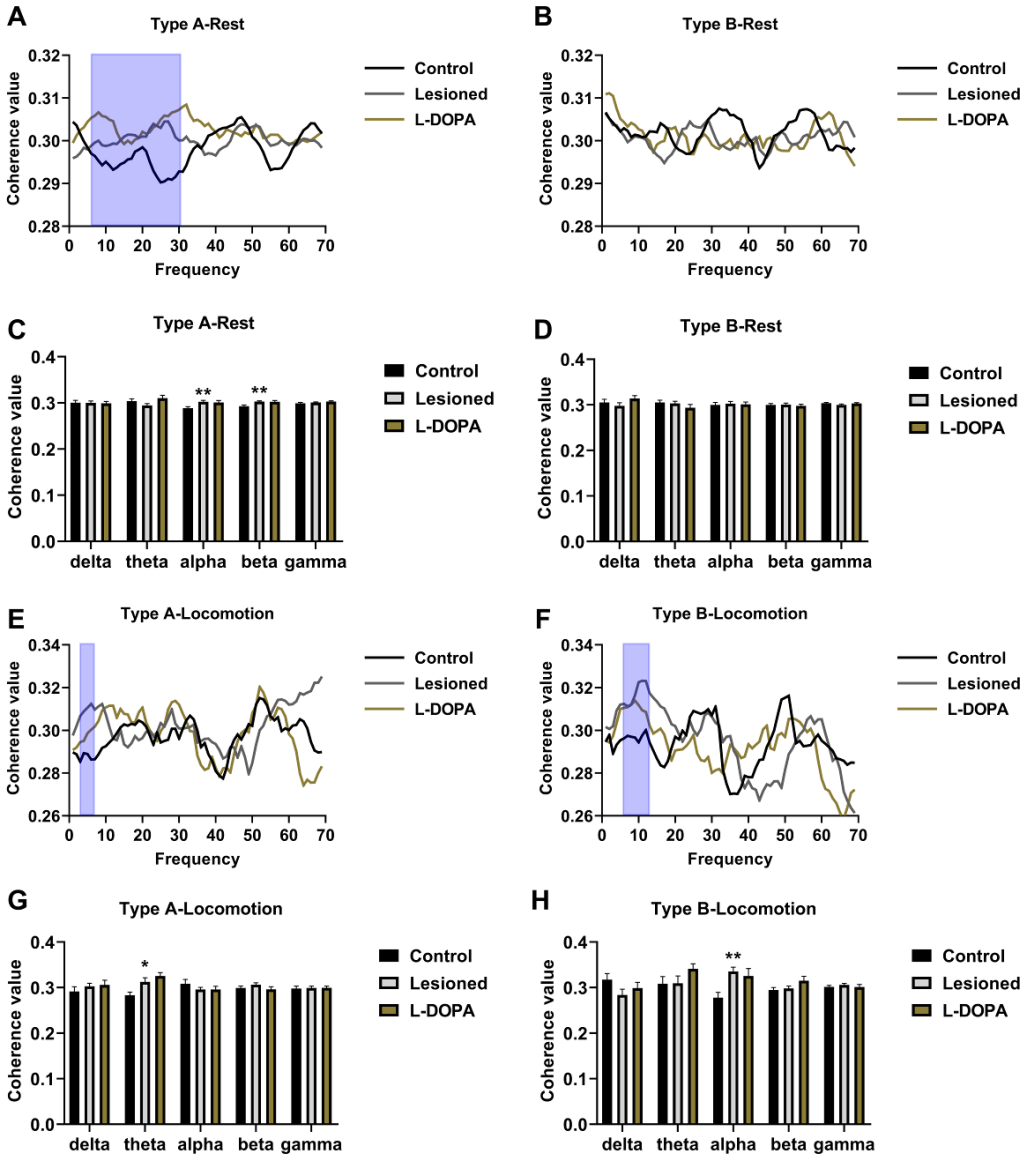
**Measurement of the coherence value between spikes and local field potential (LFP) in the pedunculopontine nucleus (PPN).** (**A**) Frequency-coherence value plots in resting Type A neurones. (**B**) Frequency-coherence value plots in resting Type B neurones. (**C**) Statistical results of coherence value in resting Type A neurones. (**D**) Statistical results of coherence value in resting Type B neurones. (**E**) Frequency-coherence value plots of Type A neurones in locomotion state. (**F**) Frequency-coherence value plots of Type B neurones in locomotion state. (**G**) Statistical results of coherence value obtained in Type A neurones in locomotion state. (**H**) Statistical results of coherence value obtained in Type B neurones in locomotion state. The frequency bands with the most noticeable changes are highlighted via blue boxes. *p < 0.05, **p < 0.01 in comparison to the control group.

To evaluate the effect of 6-OHDA lesions and L-DOPA treatment on the correlation between LFP and spiking activities in the PPN, we analysed the coherence value, SFC, and phase-lock. As shown in Figure 4A–4D, alterations in PPN Type A neurones in the resting state were detected in alpha (F (2,117) = 5.011, p = 0.0082) and beta frequency bands (F (2,117) = 5.879, p = 0.0044): 6-OHDA (n = 54) led to significantly increased coherence values between PPN Type A spikes and LFP in alpha (7–12 Hz) (p = 0.0081) and beta (12–30 Hz) (p = 0.0052) frequency bands compared to controls (n = 36). Notably, treatment with L-DOPA (n = 30) failed to reverse these changes (p = 0.9401, 0.9991 compared to in the lesioned group).

In the locomotion state (Figure 4E–4H), PPN Type A neurones in the theta band (F (2,68) = 6.245, p = 0.003), and PPN Type B neurones in the alpha band (F (2,47) = 6.347, p = 0.0047) showed altered coherence values between groups. The coherence value of the theta band was higher in the lesioned group (n = 29) than in the control group (n = 22, p = 0.0329). L-DOPA administration had no effect (n = 20, p = 0.5080 compared to the lesioned group). In addition, the coherence value of lesioned Type B neurones in the alpha band (n = 18, p = 0.0043) was higher compared to that of control rats (n = 17), and L-DOPA did not reverse this effect (n = 15, p = 0.8441 compared to lesioned rats).

### SFC

**Figure 5:**
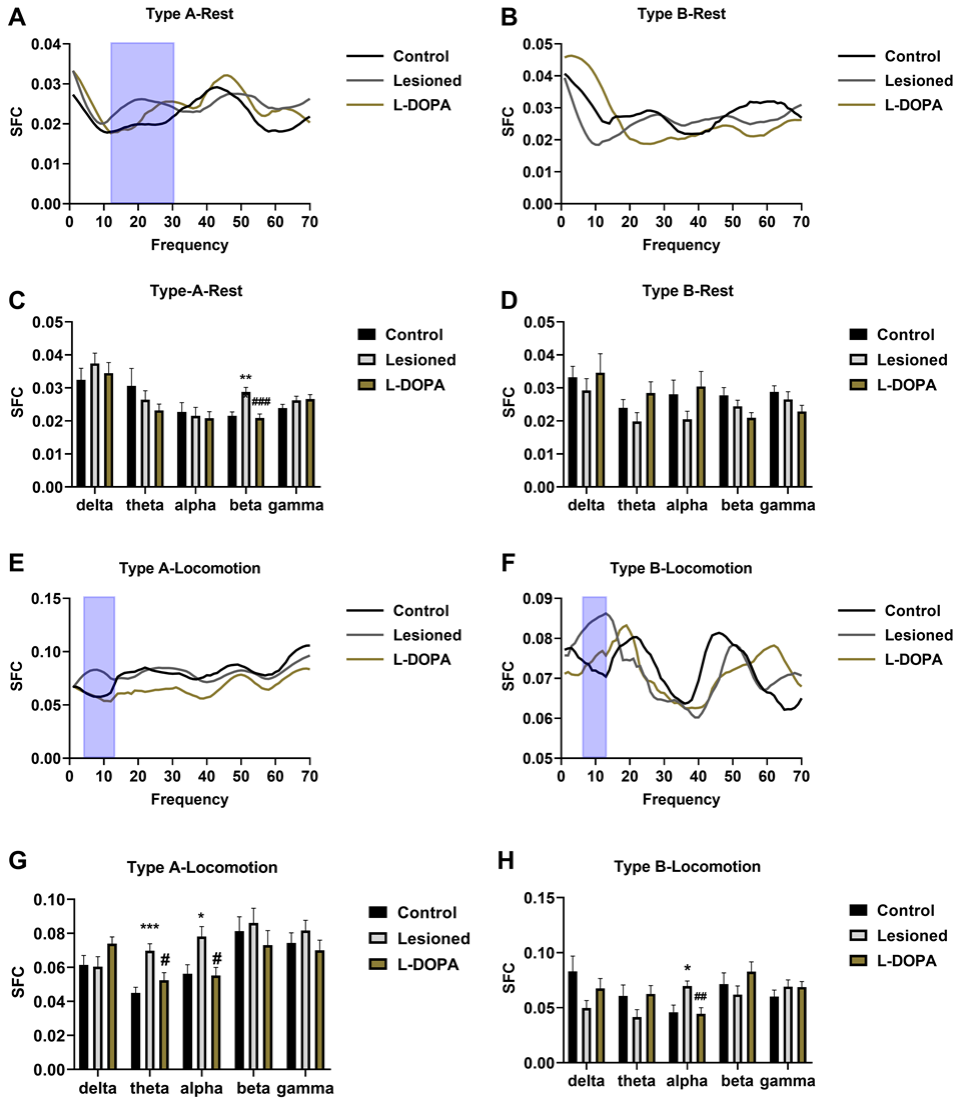
**Spike-field coherence (SFC) in the pedunculopontine nucleus (PPN) and local field potential (LFP).** (**A**) Frequency-SFC plots in resting Type A neurones. (**B**) Frequency-SFC plots in resting Type B neurones. (**C**) Statistical results of SFC obtained with resting Type A neurones. (**D**) Statistical results of SFC obtained with resting Type B neurones. (**E**) Frequency-SFC plots in Type A neurones of rats in locomotion state. (**F**) Frequency-SFC plots in Type B neurones of rats in locomotion state. (**G**) Statistical results of SFC obtained with Type A neurones of rats in locomotion state. (**H**) Statistical results of SFC obtained with Type B neurones of rats in locomotion state. The frequency bands with significant changes are highlighted via blue boxes. ***p < 0.001, *p < 0.05, relative to the control group; ###p < 0.001, ##p < 0.01, #p < 0.05, relative to the lesioned group.

Regarding the SFC in the resting state (Figure 5A–5D), Type A neurones in the beta frequency band showed differences between groups (F (2,117) = 11.942, p = 0.0003). Lesioned rats had a higher SFC than control rats (p = 0.0005), and L-DOPA treatment reversed this effect (p = 0.0002 compared with the lesioned group). In the locomotion state (Figure 5E–5G), significant differences were detected by one-way ANOVA in Type A neurones in the theta (F (2,68) = 10.415, p = 0.0009) and alpha bands (F (2,68) = 5.934, p = 0.0041) and Type B neurones in the alpha band (F (2,47) = 6.525, p = 0.0037). 6-OHDA increased the SFC of Type A neurones in the theta (p = 0.0007) and alpha bands (p = 0.0153) and SFC of Type B neurones in the alpha band (p = 0.0104). These changes were reversed upon L-DOPA administration (p = 0.0113, 0.0134, and 0.0081, respectively, compared to in lesioned rats).

### Phase-lock

**Figure 6:**
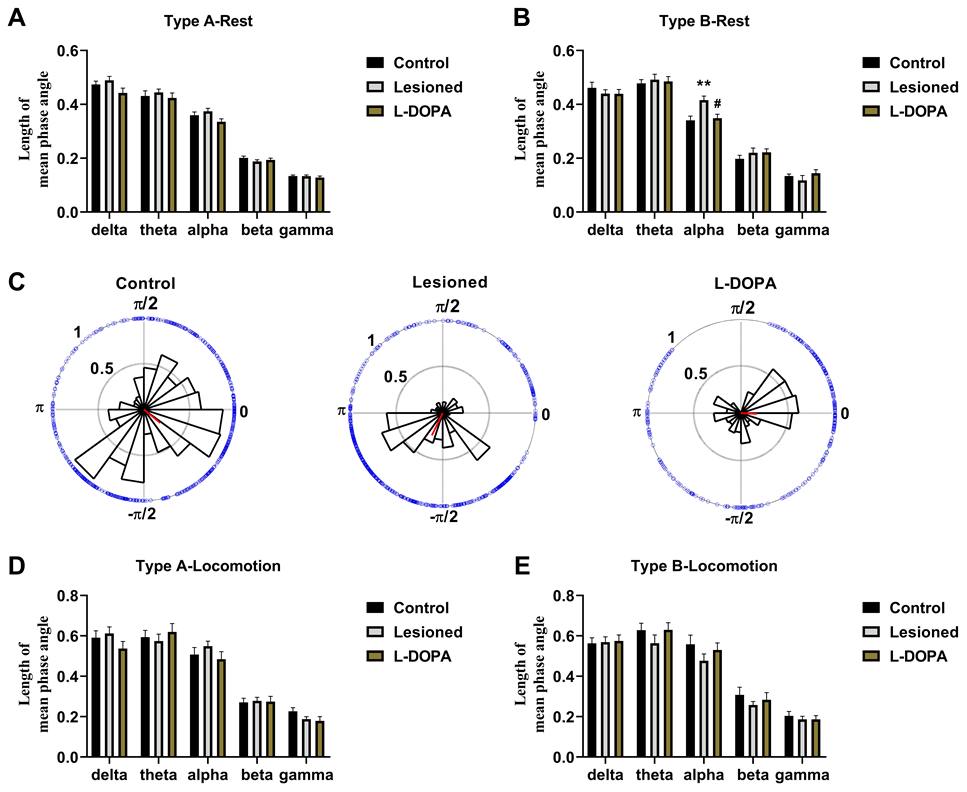
**Phase-lock between spikes and local field potential (LFP) in the pedunculopontine nucleus (PPN).** (**A**) Statistical results of phase-lock obtained with resting Type A neurones. (**B**) Statistical results of phase-lock obtained with resting Type B neurones. (**C**) Rose histograms representing the distribution of spike phase angles with oscillation in noticeably altered bands. The circles around the histograms display the phase orientation for each discharge, and the red lines arising from the centre indicate the length and vector of the mean phase angles. (**D**) Statistical results of phase-lock obtained with Type A neurones of rats in locomotion state. (**E**) Statistical results of phase-lock obtained with Type B neurones of rats in locomotion state. **p < 0.01 relative to control rats; #p < 0.05 relative to lesioned rats.

One-way ANOVA of the phase-lock data (Figure 6) revealed significant differences among resting Type B neurones of rat groups in the alpha band (F (2,54) = 7.537, p = 0.0012). Particularly, neurones in lesioned rats (n = 20, p = 0.0021) showed a higher length of mean phase angle (indicating a higher phase relationship between spikes and LFP) compared to those of control rats (n = 21). This difference was partially abolished upon L-DOPA treatment (n = 16, p = 0.0109). Figure 6 shows representative histograms illustrating the distribution of phase angles in the LFP frequency bands with significant alterations. The circles around the Rose histograms represent the phase orientation of each discharge.

## DISCUSSION

In this study, the effect of 6-OHDA lesions and L-DOPA treatment on the spike-LFP relationship in the PPN was explored using a multichannel electrical signal acquisition system. The results indicated the following: (1) the spike-LFP relationships were significantly increased in specific frequency bands by 6-OHDA lesions but differed depending on the state (rest or locomotion) and type of the neurones (Type A or Type B); (2) L-DOPA significantly affected 6-OHDA-induced SFC and phase-lock alterations. However, L-DOPA administration failed to restore the coherence value, highlighting the potential limitations of L-DOPA in PD therapy.

Coherence, SFC, and phase-lock analyses allowed for comprehensive investigation of spike-LFP synchronisation in two distinct types of PPN neurones. LFP is thought to reflect overall transmembrane currents within the local region around the electrode and is measured as a rhythmic activity in different frequency bands. It can be used to synchronise neuronal spiking activities, and spike-LFP synchronisation can be assessed using algorithms to calculate coherency, SFC, and phase-lock. Recording of electrophysiological signals and synchronisation analysis were used to assess the responses of two different types of PPN neurones to 6-OHDA-induced lesions and L-DOPA administration. Signal recording in both resting and locomotion state ensured that the physiological conditions were reproduced.

In this study, the examined PPN neurones were classified into two different types (A and B) based on our previous articles. In a recent study [11], PPN signals were recorded in patients with PD and, similar to in our study, neurones were classified into two types. The PPN has heterogeneous neurochemistry characteristics as it contains cholinergic and noncholinergic (γ-aminobutyric acid and glutamatergic) neurones and forms diverse projections with other brain regions such as the motor cortex, globus pallidus, SN, thalamus, subthalamic nucleus, etc. Previous studies showed that the posterior part of the PPN mainly contained cholinergic neurones [21]. The neuron firing waveforms with a wide duration and low firing rate (Type B in this research) were considered as cholinergic neurons by juxtacellular labelling and immunofluorescence identification [16]. Cholinergic neurons in the PPN are thought to be highly correlated with movement, as they are vital for relaying sensory information from the spinal cord to the thalamus and SNc, and they can radiate downward and terminate in the pons and midbrain [22]. Additionally, neurone firing waveforms with a narrow duration and high firing rate (Type A in this research) were presumed to be noncholinergic neurones.

In contrast, we classified neurones based on their electrophysiological properties but no further neurochemical characterisation was performed, nor was morphological evaluation of PPN neurone conducted to support the electrophysiological data. These factors require further examination. Moreover, because of the size limitation of the recording electrode array, we could not distinguish between the anterior subregion of the PPN, preferentially projecting to the substantia nigra, and posterior portion, projecting to the ventral tegmental area [23]. Studies are needed to evaluate the heterogeneous characteristics of PPN and explore the clinical applicability of DBS in the PPN [24, 25].

Although evidence for changes in PPN spike-LFP synchronisation following 6-OHDA lesions and L-DOPA administration has rarely been reported, many studies demonstrated that alterations in electrophysiological activities occur in the PPN in PD [12, 14, 16, 17, 26–29]. Neuronal loss in the PPN of patients with PD was reported previously [30]. However, the specific locomotion states and changes in the spike-LFP relationship with PD related to neuronal activity remain unclear. Our results indicate that in the PPN, the spike-LFP relationship was significantly affected by 6-OHDA lesions in specific frequency bands; this phenomenon may be explained by the tight connections between the PPN and basal ganglia. The PPN is the output and input region for the basal ganglia and a key regulator of the thalamus (and, consequently, the cortex) [23]. The PPN receives inhibitory projections from the globus pallidus interna and substantia nigra pars reticulata, as well as excitatory projections from the subthalamic nucleus. Therefore, the activity of PPN neurones is significantly affected by extensive pathological changes in the basal ganglia [8, 9, 31, 32].

Our understanding of brain function is based on the recording of extracellular signals utilising electrodes implanted in specific brain regions, which provides a direct measure of electrical activity consisting of spikes and LFP. Spikes are single-unit activities emitted by one or more neurones around the electrode tips, whereas LFP represents the total synaptic current [33, 34]. The spike and LFP signals recorded by the same electrode reflect the responses of a single neurone to large-scale circuit activities. Common methods for analysing spike-LFP relationships can be divided into time domain (such as the SFC and phase-lock) and spectral-domain (such as coherence) methods [35]. Band-specific alterations in the spike-LFP relationship were previously used to investigate the mechanism of neural coding [36].

Overall, the combined examination of coherence value, SFC, and phase-lock revealed some inconsistencies between these algorithms. Specifically, no difference between groups was observed in the mean length of phase angle in Type A cells during locomotion, and SFC and coherence values were significantly altered following 6-OHDA lesions. Similarly, in lesioned rats, Type B cells exhibited significant changes in coherence and SFC in the alpha frequency during locomotion but no change was observed in the mean phase angle length under the same conditions. The same inconsistency was observed in our previous research of the spike-LFP relationship in the PPN and motor cortex [37]. The differences in the results obtained with the three algorithms may be explained as follows: the coherence value is a function of the frequency domain, which is equivalent to the time domain [38]. The phase-lock analysis explores the angular distribution of spikes in relation to LFP oscillation [20]. The SFC is the ratio of the spike-triggered and the LFP traces the average frequency spectrum, as well as includes information from both the LFP spectrum and spike activities [18]. The above differences between algorithms or formulas may explain the inconsistencies in our results.

L-DOPA, the first-line drug in PD therapy, reverses abnormal activity in disease-affected brain areas [4, 39–41]. Our previous study demonstrated the effect of L-DOPA on the association between the PPN and motor cortex [42]. However, the impact of L-DOPA on spike-LFP synchronisation in the PPN has not been analysed, and no studies have been conducted in freely moving animals. We explored the effects of L-DOPA on the spike-LFP relationship of PPN neurons. Notably, although L-DOPA improves most clinical PD symptoms, it does not attenuate gait and posture symptoms [3], further supporting our hypothesis that L-DOPA has limitations such as the spike-LFP coherence value in PPN. Moreover, for the refractory gait and posture symptoms, PPN DBS has significant therapeutic effects, as it is a key region in the midbrain locomotor region, supporting our hypothesis [6]. Our results suggest that PPN DBS reverses the gait and postural symptoms in PD by balancing the amplitude between spikes and LFP; however, further studies of PPN function are needed to verify this possibility.

In conclusion, our findings reveal specific changes in spike-LFP relationships after dopamine loss and L-DOPA treatment in both resting and locomotion-state rats, suggesting that 6-OHDA raises coherency and phase-lock activities in a specific frequency band, and highlight a possible limitation of L-DOPA therapy. Our study provides evidence for the role of PPN in the pathogenesis of PD and may provide a basis for clinical treatment of this refractory disease.

## MATERIALS AND METHODS

### Subjects

All experiments were conducted using male adult Wistar rats (280–320 g, purchased from Vital River Laboratories, Beijing, China), which were housed at 21 ± 1° C and 55% relative humidity under a 12:12-h light-dark cycle and provided with water and food *ad libitum* for at least 7 days before use. All experiments were approved by the ethics review board of Shandong University of Traditional Chinese Medicine (No. AE-20180905-01). All efforts were made to minimise the pain of animals as per the guidelines of the National Institute of Health for the Use and Care of Laboratory Animals and Animal Experiment Management Regulations approved by Shandong University of Traditional Chinese Medicine. The animals were separated into a control group (rats with saline injection in the medial forebrain bundle, MFB), lesioned group (rats with 6-OHDA injection in the MFB), and L-DOPA group (rats with 6-OHDA injection in the MFB and L-DOPA treatment).

### Walking training

A ladder (100 × 20 cm) was employed for walking training as previously described [17]. The rats were fasted for 24 h and kept at one end of the ladder. A food reward was used to train rats to walk to the opposite end automatically. The rats were trained three times per day for 3 days. After training, the rats exhibited stable and consistent responses and could walk without pausing during electrophysiological signal recording. On the day of signal recording (29th day of the experimental timeline), the walking time of each rat was calculated and used as an indicator of motor function.

### 6-OHDA lesion and apomorphine-induced rotation test

To establish a rat model of PD, rats in the lesioned and L-DOPA groups were subjected to unilateral 6-OHDA lesion surgery [37]. The rats were anaesthetised with 4% chloralhydrate (360 mg/kg, intraperitoneally [i.p.]) and mounted in a stereotaxic frame. A burr hole was drilled above the left MFB (2.2 mm posterior and 2.1 mm lateral to the bregma), and 3 μL of 6-OHDA (4 μg/μL dissolved in 0.9% sodium chloride and 0.02% ascorbic acid; Sigma, St. Louis, MO, USA) was vertically injected 8.53 mm ventral to the surface of the skull at a rate of 1 μL/min using a microsyringe. To favour diffusion, we retained the syringe at the injection site for approximately 10 min, and then gradually withdrew it. Control rats underwent sham operation with injection of 3 μL of solvent. Suturing was performed at the incision, and the rat was administered 0.5 mL of penicillin (160 mg/kg, i.p.) each day for 3 days to avoid infection. Fourteen days after surgery (on day 14 of the experimental timeline), rats in lesioned and L-DOPA groups were injected with 0.5 mg/kg apomorphine (APO; Sigma) intramuscularly to induce contralateral rotation. The PD model was considered as successful if the rat performed seven turns per min within 30 min after injection. Only rats with successful lesions were included in the lesioned and L-DOPA groups. On day 29 of the experimental timeline (after treatment with L-DOPA), the above-described test was repeated to evaluate the drug effect.

### L-DOPA treatment

To avoid the side effects of drugs that are common in clinical or animal research [40], we chose a low-dose, long-term regimen. Rats in the L-DOPA group were treated daily with 4.5 mg/kg L-DOPA (i.p.; Sigma) combined with 4.5 mg/kg benserazide (i.p.; Sigma) for 14 days. The drugs were dissolved in 0.5 mL of 0.9% sodium chloride with 0.02% ascorbic acid and administered three times daily (1.5 mg/kg L-DOPA and 1.5 mg/kg benserazide each time), i.e., at 9:00, 15:00, and 21:00 h [42]. Rats in the control and lesioned groups were mock-injected with solvent. No L-DOPA-induced dyskinesia was observed throughout the experiment.

### Electrode implantation

During days 20–22 of the experimental timeline, a multi-unit recording electrode was implanted in all rats. The animals were anaesthetised with chloralhydrate (360 mg/kg, i.p.) and mounted in a stereotaxic frame. A home-made 16-channel electrode (consisting of 16 nickel-chromium Teflon-insulated microwires) was mounted on a stereotaxic frame into the rat PPN with the following coordinates: 1.9 mm lateral and 7.8 mm posterior to bregma, and 7.3 mm ventral to the skull surface, ipsilaterally to the 6-OHDA lesion surgery. The diameter of each microwire (purchased from California Fine Wire, Grover Beach, CA, USA) was 25 μm, and the tip impedance was 0.3–0.7MΩ at 1004 Hz. The Hong Kong Plexon Company provided guidelines for fabricating the electrodes. The Teflon was removed from one end of the microwire and welded to the socket. The other end was converged in a bundle of electrodes (with a cross-sectional area of 0.15 × 0.15 mm^2^ and an array of 4 × 4) to be placed in the PPN. Considering the shadowing effects of single-unit recordings in adjacent electrodes, if the recordings collected from two adjacent electrodes were consistent in all parameters, one of the channels was removed from the analysis. The brain tissue was covered with melted agarose, and the ground wire of the electrode was bound tightly around the stainless-steel screws placed on the skull. The signal displayed in the process of electrode implantation provided stereotaxic assistant and guidance for the electrode position. Finally, the dental cement was used to fix the electrode to the skull, and the rat was administered 0.5 mL of penicillin (160 mg/kg, i.p.) every day for 3 days to prevent infection.

### Rotarod test

To assess the beneficial effects of 6-OHDA lesion and L-DOPA on the locomotor function of rats, we performed a rotarod test using a rotarod treadmill (rod diameter: 64 mm, UGO Basile, Gemonio Varese, Italy) three times on day 29 of the experimental timeline at a constant speed (20 rounds per min) for 30 min. The longest latency and distance obtained with each animal were used for statistical analysis.

### Signal recording

The electrophysiological signals were recorded on day 29 of the experimental timeline. Extracellular signals, i.e., spikes (sample rate 20 kHz, band-pass filtering 300–8000 Hz) and LFP (sample rate 1 kHz, band-pass filtering 0.5–200 Hz) were obtained using the 16-channel OmniPlex D Neural Data Acquisition System (Plexon, Dallas, TX, USA) in rats in resting (awake but not paying attention to their environment) and locomotion (ladder-walking) state. Videos were used to monitor animal conditions and recorded synchronously. The basal noise of the wideband (0.5–8000 Hz) signals indicated animal state differences compared to under resting conditions. Spike signals were recorded only when the signal-to-noise ratio was >2. During locomotion, the signals recorded throughout the walking task were extracted for analysis. Signal analysis was performed using Offline Sorter V4 (Plexon), Neuroexplorer V5 (Nex Technologies, Nays, KS, USA), and MATLAB (Mathworks, Natick, MA, USA).

### Histological staining

On day 30 of the experimental timeline, the rats were sacrificed with 4% chloralhydrate (i.p.). An electrical microlesion (10 μA, 10 s × 5) was induced via an anodal current through the electrodes. The brains were collected after intracardial perfusion with 0.01 M phosphate-buffered saline (PBS) and paraformaldehyde (4%) containing potassium ferricyanide (1%). After post-fixation in 4% paraformaldehyde, the brains were dehydrated in 30% sucrose. Frozen sections were obtained, and 40-μm coronal sections encompassing the PPN were collected. The sections were stained with cresyl violet to examine the tracks and sites of electrodes, and only animals with correct electrode positioning were used for data analysis.

To determine the level of dopaminergic neurone degeneration, we collected sections encompassing the SNc of animals that underwent 6-OHDA injection surgery, and half of of the sections were stained with cresyl violet. The remaining sections were examined via histochemical tyrosine hydroxylase staining. The sections were washed with 0.01 M PBS, incubated for 6 h with blocking buffer containing Triton X-100 (0.5%) and normal donkey serum (5%), and then incubated with the primary polyclonal rabbit antibody (1:1000; ab6211; Abcam, Cambridge, UK) at 4° C overnight. The sections were incubated with fluorescein isothiocyanate-conjugated donkey anti-rabbit IgG (1:200; sc-2090; Santa Cruz Biotechnology, Dallas, TX, USA) for 2 h, washed with PBS, mounted with antifading mounting medium (S2100; Solarbio, Beijing, China), and the coverslips were fixed with nail polish. The primary and secondary antibodies were diluted with 0.01 M PBS containing Triton X-100 (0.3%) and donkey serum (3%). For the negative control, the same procedure was followed but the primary antibody was omitted. The sections stained with cresyl violet were observed, and the images were captured with an optical microscope (Axio Lab A1; Zeiss, Oberkochen, Germany) and tyrosine hydroxylase-stained sections were captured with a laser confocal microscope (Leica TCS SPE; Wetzlar, Germany).

### Data analysis

### Neurone classification

The recorded signals were imported into Offline Sorter Software, and cross-channel artefacts and waveforms with short ISIs (less than 2 ms) were eliminated with the built-in function. Using the k-means clustering algorithm in PCA, the waveforms in each channel were sorted. Waveforms with analogous characteristics were gathered as a cluster in the 2D/3D view. Two separate clusters were defined as different neurones after application of the F statistic parametric multivariate ANOVA (analysis of variance, p < 0.05). Manual checking was conducted to guarantee that spurious signals were eliminated. The characteristics of each neurone in the PPN were examined, and the spikes of each neurone were identified based on the waveform shape, duration of peak-to-trough, inter-spike interval histograms, PCA analysis, and firing rate [17].

Six signal segments per neurone in resting or locomotion state were randomly selected according to the random digits method. The neurone mean level was defined as the mean value of these six segments. The length of each segment was 10 s in resting state and corresponded to the time of walking along the ladder in the locomotion state.

### Coherence analysis

**Figure 7:**
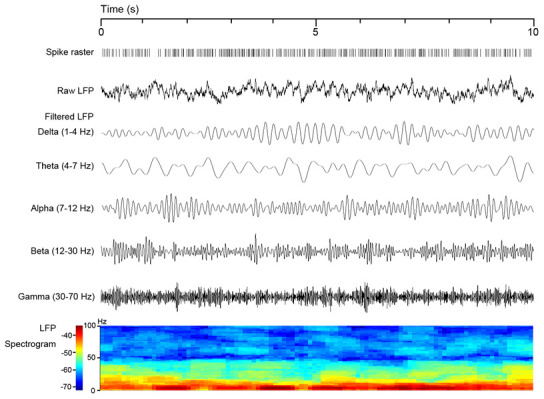
**Local field potential (LFP) filtering process is illustrated as a representative 10-s signal in resting state with synchronously recorded spike raster and an LFP spectrogram.**

The selected signals were imported into MATLAB, and analysis was conducted using the open-source data analysis toolbox Chronux 2.0 (http://chronux.org) and CircStat Toolbox [43]. To investigate the effect of the 6-OHDA lesion and L-DOPA treatment on the spike-LFP synchronisation of PPN, we applied three algorithms for coherence, SFC, and phase-lock analyses according to our previous studies [16, 37]. The LFP signals were separated into five bands: delta, 1–4 Hz; theta, 4–7 Hz; alpha, 7–12 Hz; beta, 12–30 Hz; gamma, 30–70 Hz. The spike-LFP synchronisation of each neuronal type in the PPN was analysed in different LFP frequency bands. In Figure 7, the LFP filtering process is shown as a representative of the 10-s signal in the resting state, and the synchronously recorded spike raster indicates single-unit activities. In addition, an LFP spectrogram obtained with the fast Fourier transfer-based log of power spectral density method with multiple tapers (3 time-bandwidth and five tapers) is shown.

With the coherence function in Chronux, a multitaper method with 0.5-s window size, 0.1-s time interval, nine tapers, five time-bandwidth and 1 kHz frequency sample were used in coherence analysis. Variations in the values of coherence between zero to one were calculated, and zero indicated that the spike and LFP had no relation, whereas one indicated a perfect correlation.

### SFC analysis

In this study, SFC analysis was performed to evaluate the timing relationship between oscillations and spikes. The SFC was calculated as the ratio between the frequency spectrum of spike-triggered average (STA) and the average spike-triggered power (STP) frequency spectrum of the LFP traces in specific frequency bands: SFC = (fSTA/STP) × 100% [18]. The STA was obtained by extracting the unfiltered LFP signals (centred on each spike ± 500 ms), and the STA spectrum was calculated (3 time-bandwidth, five tapers, window overlap of 50%). For each signal segment, the number of spikes was equalised to 50 and 20 in resting state and locomotion state, respectively.

### Analysis of phase-lock

The Rayleigh test was performed to assess whether the spikes were noticeably phase-locked with LFP at specific frequency bands. Only segments with p < 0.05 were used for further analysis. As the spikes and LFP were recorded synchronously, spikes occurring at LFP oscillation peaks were considered as 0° C phase, whereas spikes at troughs were considered as 180° C (π) or -180° C (-π) phase. Subsequently, using the CircStat Toolbox, the mean phase angle and resulting vector length were calculated. The mean phase angle length varied from 0 to 1. When the mean phase angle length approached 1, the neurone showed a greater correlation with the LFP oscillations because the angles were more concentrated around the mean phase angle. The mean phase angle vector and length were plotted in a Rose histogram.

### Statistics

SPSS software 23.0 (SPSS, Inc., Chicago, IL, USA) was used for statistical analysis, and the data were represented as the mean ± standard error. The Kolmogorov-Smirnov test was used to evaluate the normality of data distribution. The differences between rat groups (control, lesioned, and L-DOPA) were assessed by one-way ANOVA followed by Tukey’s post hoc comparison. For the APO-induced rotation, a two-tailed unpaired *t*-test was performed between two groups. The threshold for statistical significance was set at p < 0.05, and the level of significance was defined as *p < 0.05, **p < 0.01, ***p < 0.001, and ****p < 0.0001.
